# Selectively Interfering With Intrusive but Not Voluntary Memories of a Trauma Film: Accounting for the Role of Associative Memory

**DOI:** 10.1177/2167702621998315

**Published:** 2021-05-06

**Authors:** Alex Lau-Zhu, Richard N. Henson, Emily A. Holmes

**Affiliations:** 1 Medical Research Council Cognition and Brain Sciences Unit, Department of Psychiatry, University of Cambridge; 2 Oxford Institute of Clinical Psychology Training and Research, Division of Medical Sciences, University of Oxford; 3 Centre for Psychiatry, Department of Brain Sciences, Imperial College London; 4 Oxford Health NHS Foundation Trust, Oxford, England; 5 Department of Psychology, Uppsala University; 6 Department for Clinical Neuroscience, Karolinska Institutet

**Keywords:** intrusive memories, involuntary memory, mental imagery, memory consolidation, trauma, PTSD, open data, open materials

## Abstract

Intrusive memories of a traumatic event can be reduced by a subsequent interference procedure, seemingly sparing voluntary memory for that event. This *selective-interference effect* has potential therapeutic benefits (e.g., for emotional disorders) and legal importance (e.g., for witness testimony). However, the measurements of intrusive memory and voluntary memory typically differ in the role of associations between a cue and the emotional memory “hotspots.” To test this, we asked participants to watch a traumatic film followed by either an interference procedure (reminder plus Tetris) or control procedure (reminder only). Measurement of intrusions (using a laboratory task) and voluntary memory (recognition for film stills) were crossed with the presence or absence of associative cues. The reminder-plus-Tetris group exhibited fewer intrusions despite comparable recognition memory, replicating the results of prior studies. Note that this selective interference did not appear to depend on associative cues. This involuntary versus voluntary memory dissociation for emotional material further supports separate-trace memory theories and has applied advantages.

In autobiographical memory research, an important distinction is made between *voluntary* memory, which involves remembering with deliberate intent, and *involuntary* memory, which involves recollecting the event spontaneously (Berntsen, 2009; Conway & Pleydell-Pearce, 2000). Involuntary memories are also considered “intrusive” when they are recurrent and unwanted (Berntsen & Rubin, 2014; Kvavilashvili, 2014). Intrusive memories occur across many emotional disorders (Brewin et al., 2010; Holmes & Mathews, 2010; Kavanagh et al., 2005). Traumatic events, in particular, tend to lead to intrusive memories, which are typically image-based (Arntz et al., 2005; Ehlers et al., 2004). For instance, a memory “hotspot” of the moment when two cars collide during a road traffic accident may later intrude unbidden, vividly in the mind’s eye, bringing distress (Iyadurai et al., 2019). It is this involuntary quality of these images that is associated with the core clinical feature of posttraumatic stress disorder (PTSD; American Psychiatric Association, 2013)—intrusive memories of a traumatic event.

The theoretical distinction between intrusive and voluntary memory of trauma holds clinical importance. It is increasingly recognized that the development of memory therapeutics must consider carefully which types of memory are and are not modified (Elsey & Kindt, 2016; Phelps & Hofmann, 2019; Visser et al., 2018). For example, one may seek to reduce the occurrence of distressing intrusions in therapy but without inadvertently compromising deliberate recall of the same trauma (Holmes, Sandberg, & Iyadurai, 2010), which can be important. Trauma survivors may need to be able to voluntarily describe the details of their trauma(s) for the purpose of legal testimony. Moreover, amnesia resulting from an inability to voluntarily retrieve important aspects of trauma (e.g., after a drug rape) can be associated with clinical distress (Gauntlett-Gilbert et al., 2004).

Experimental data suggest that intrusive memory and voluntary memory for the same material are dissociable (e.g., Deeprose et al., 2012; Hørlyck et al., 2019; Holmes et al., 2009; Holmes, James, et al., 2010; James et al., 2015; Lau-Zhu et al., 2019). In a series of experiments using a distressing film as an experimental trauma (James et al., 2016; Lau-Zhu et al., 2018), we have established that the occurrence of intrusive memories of such films can be reduced without apparent reduction in voluntary memory for the films (e.g., as tested by recall and recognition)—a phenomenon we referred to as a *selective-interference effect* (Lau-Zhu et al., 2019). The effect resulted from administering an interference procedure soon after film viewing—here, a reminder cue (to the experimental trauma) followed by the computer game Tetris played with mental rotation instructions (i.e., reminder plus Tetris; Holmes et al., 2009; Holmes, James, et al., 2010; Lau-Zhu et al., 2019). The procedure is thought to interfere with consolidation of memory for the film (Nader, 2003) and selectively interfere with the type of memory that constitutes intrusive mental images (Brewin, 2014; Ehlers et al., 2004; Holmes et al., 2009). We found evidence that the selective-interference effect on trauma-film intrusions can be observed soon after interference—just several minutes later (Lau-Zhu et al., 2019, Experiments 2 and 3).

Selective interference can be interpreted in line with separate-trace memory theories (Dalgleish, 2004), such as a dual-representation account of emotional memory in PTSD (Bisby & Burgess, 2017; Brewin, 2014; Brewin et al., 1996). A recent version of this theory posits that in addition to the ordinary autobiographical memory system that subserves voluntary retrieval, a functionally independent, long-term, image-based system subserves intrusive memories in PTSD. Such an account contrasts with the dominant view in cognitive psychology that a single memory system underlies declarative (conscious) memory (Schacter & Tulving, 1994) regardless of whether memory is expressed voluntarily or involuntarily (Berntsen, 2009; Tulving, 2002). According to this single-trace perspective, interfering with memory consolidation should affect both forms of retrieval rather than interfere selectively with only involuntary (intrusive) memories.

Claims that a postencoding interference selectively affects different types of memory, however, demand careful methodological examination of how memory is measured. In trauma-film research (James et al., 2016; Lau-Zhu et al., 2018), intrusive memories have been typically measured by a pen-and-paper diary recorded during everyday life over several days after watching the film, and voluntary memory has been typically assessed with a recognition memory task performed in the laboratory 1 week later (requiring yes/no judgments for scenes from the trauma films vs. scenes from unseen films). Diaries and recognition tasks come from different research traditions and are vastly different methodologically. Hence, differential effects on these two memory measures may not necessarily reflect a dissociation between involuntary and voluntary memory but, rather, other methodological factors, including the nature of the cues, attentional demands (e.g., what people are doing when intrusions arise), and the retention interval. Recently, we matched several such factors yet still found selective interference from a cognitive procedure (reminder plus Tetris) on intrusions but not on voluntary memory across three experiments (Lau-Zhu et al., 2019).

One key factor that has not yet been addressed when studying intrusive and voluntary memory comparisons, however, is the role of associative memory. That is, previous measures of intrusive memories seem to include an associative component requiring memory for associations between elements within a traumatic event (e.g., the scenes in a distressing film), whereas previous measures of voluntary memory normally do not. Because intrusive memories are typically recorded in diaries—with instructions primarily given to report what “pops into mind” (James et al., 2016)—it may seem strange to characterize the diary as a task involving an associative component. Yet we have found that participants were often able to self-report cues from their everyday environment that had seemingly triggered their trauma-film intrusions (Lau-Zhu et al., 2019; Experiments 1 and 2). As an example of a trigger-hotspot link in the diary task, “seeing a child cross the road” in everyday life could have triggered an intrusive image of the film in which “a school boy is crashed by a van while texting and crossing the road.” The role of associative cues in triggering intrusions is acknowledged in clinical models of PTSD (Ehlers & Clark, 2000; Foa & Kozak, 1986), such as seeing “a red post” may trigger intrusions of “a car crash involving a red car.”

Inspired by observations of cues in the diary—alongside existing intrusion provocation approaches in the lab (Lau-Zhu et al., 2018)—we developed a laboratory analogue of the diary, which we called the *vigilance-intrusion task* (Lau-Zhu et al., 2019, Experiments 2 and 3). In this task, participants perform a simple vigilance task (i.e., a go/no-go task on a series of numbers) designed to capture the attentional demands of typical everyday tasks (e.g., reading or shopping) while initially unattended cues, designed to trigger intrusive memories of a prior trauma film, are presented occasionally in the background. While performing this task, a background still depicting an initial scene with “a child happily playing football” might trigger an intrusion from an associated hotspot (i.e., worst moment; Holmes et al., 2005) within the film when “the child is later killed by a car.” Such trigger-hotspot links (or cue-target links) can be conceived as a form of associative memory—that is, memory for the associations between different scenes—rather than memory for the scenes themselves (Yonelinas, 2002).

Measurement of voluntary memory in trauma-film studies has tended to rely on single-item recognition tests (e.g., James et al., 2015, Experiment 2; Lau-Zhu et al., 2019, Experiments 1 and 2), which we reason less likely involve an associative component. In visual/verbal recognition memory tests, a cue is presented either describing or depicting a particular scene of the film, for example, a hotspot moment of “the car crash killing the child.” The cue provided is tested for its content but without necessarily testing its association with other scenes (e.g., of “the child playing football prior to the crash”). Indeed, recognition for single stimuli can be achieved by a feeling of familiarity without any (associative) recollection of other information (Yonelinas, 2002). We therefore propose that associations are better probed in the intrusion measures, such as the vigilance-intrusion task, because the provoked intrusive hotspots memories are different in content to what is depicted in the neutral cues themselves. Thus, it is possible that the interference procedure (reminder plus Tetris) disrupts the consolidation of associations between elements within a traumatic event (which in turn reduces the probability of a cue triggering an intrusion) without affecting consolidation of the individual elements (as tested in traditional voluntary tests of recognition memory). It may appear that voluntary memory is preserved, whereas in fact, the associations have been eroded.

## Overview of the Current Experiment

In the present study, we tested whether there is indeed a selective-interference effect or whether measurements of intrusive and voluntary memory of a trauma film differ in whether an associative component is included. We used the vigilance-intrusion task together with a visual-recognition memory task but, critically, modified both to more clearly distinguish conditions with and without an associative component (via the presence and absence of associative cues). As in Lau-Zhu et al. (2019, Experiments 1–3), participants were randomly assigned to a cognitive-task-interference group (reminder plus Tetris) or a control no-interference group (reminder only) 30 min after they viewed a trauma film. A pre-Tetris reminder was included in a successful real-world clinical translation of this interference procedure (Iyadurai et al., 2018; Kanstrup, Singh, et al., 2021), critically shown to be necessary in the laboratory to observe interference on intrusions (Lau-Zhu et al., 2019). Indeed, absence of this reminder component was associated with a lack of interference effect (Brühl et al., 2019). This is presumably because a reminder cue helps bring a target memory into one’s attention sufficiently to then successfully exert interference on that memory (Visser et al., 2018).

The rationale for the 30-min postfilm delay was as follows: (a) It may fall within the putative time window of memory consolidation when a memory trace is hypothesized to remain labile (up to 6 hr after initial encoding) and amenable to change, at least according to some accounts (e.g., Nader et al., 2000); (b) it is considered an approximate time typically reported for accident and emergency services to arrive at a trauma scene in the United States (Carr et al., 2009) as well as in the United Kingdom (National Audit Office, 2017); (c) it is of the same duration as previous consolidation-based studies in this line of work, which we seek to replicate and extend (Badawi et al., 2020; Deeprose et al., 2012; Holmes et al., 2009; Holmes, James, et al., 2010; Lau-Zhu et al., 2019).

Subsequently, participants completed measures of involuntary memory (vigilance-intrusion tasks) and voluntary memory (modified recognition task) within the same experimental session on Day 1. For the vigilance-intrusion tasks to assess intrusions (while people perform a simple go/no-go task on numbers), we compared a version with trauma-film (associative) cues (as in Lau-Zhu et al., 2019, Experiments 2 and 3) and another version without such cues. For the modified recognition task, we similarly included two types of target trials (within the same task), half of which were preceded by trauma-film (associative) cues and half by cues from a different/unseen film (foils). The logic was that if trauma-film cues (e.g., a still depicting a nonintrusive/neutral scene) trigger memory for associations and enhance subsequent retrieval, then we should find increased intrusion rates and improved recognition performance. Note that measures of intrusions and recognition were matched in terms of cues (stills from films), context (both in the laboratory), attentional demands (participants performed a no/no-go task on the cues in both tasks), as well as retention interval (shortly after interference was delivered).

Following previous research (Badawi et al., 2020; Deeprose et al., 2012; Holmes et al., 2009; Holmes, James, et al., 2010; Lau-Zhu et al., 2019), baseline measures were completed at the start of the experiment on key clinical/cognitive dimensions that have been associated with increased risk for intrusive memories, including depression and anxiety (e.g., Brewin et al., 2010; Holmes et al., 2016), trauma history (e.g., Ozer et al., 2003), and mental imagery use (e.g., Kosslyn, 2005; Morina et al., 2013), to check for successful random assignments and rule these out as potential confounds.

## Aims and Hypotheses

The first aim was to attempt to replicate the interference effect (after viewing a trauma film) of the reminder-plus-Tetris procedure on subsequent intrusions of the film, as assessed in a vigilance-intrusion task embedded with trauma-film cues (on same day, i.e., Day 1, as in Lau-Zhu et al., 2019, Experiments 2 and 3), as well as on diary intrusions of the film over the subsequent week (Holmes et al., 2009; Lau-Zhu et al., 2019).

**Fig. 1.**
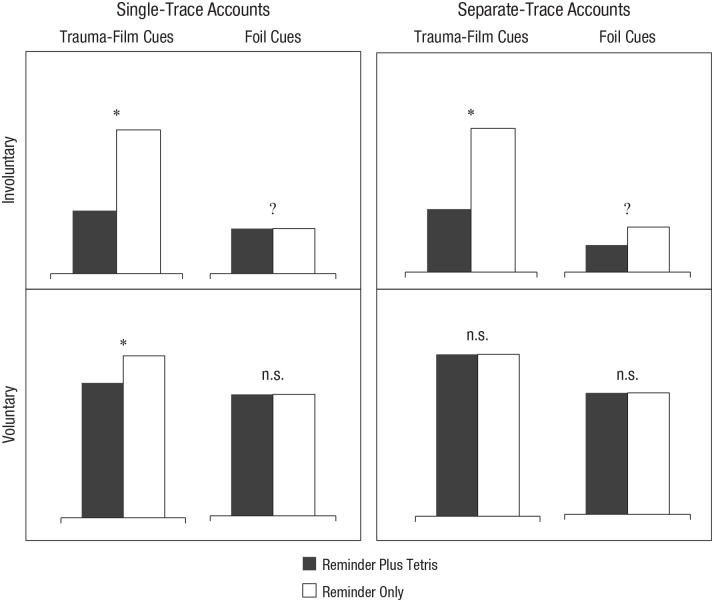
Schematic of contrasting predictions on memory outcomes by single-trace accounts compared with separate-trace accounts. n.s. = not significant. Asterisks indicate a significant difference between groups (*p* < .05). Question marks indicate an unclear pattern.

The second and critical aim was to see whether similar a pattern of disruption could be found on a voluntary recognition-memory task in which the target scenes (requiring a yes/no response) were preceded by cues either from the trauma film or a different/unseen film. See Figure 1 for a schematic of the differential predictions by each theoretical perspective. On one hand, if similar disruption were found on both involuntary and voluntary memory measures when including an associative component (i.e., with trauma-film cues), this would support the hypothesis that reminder plus Tetris interferes with associations between elements of a memory, which would be more consistent with single-trace accounts. On the other hand, if interference were found only on an involuntary memory measure (e.g., in a vigilance-intrusion task) and not on the voluntary memory measure (recognition task), this would strengthen prior claims that separate memory systems underlie voluntary and involuntary (intrusive) memories.

## Method

### Participants

Thirty-six participants (27 women, mean age = 24.33 years, *SD* = 3.41, range = 19–36) were recruited from the Medical Research Council Cognition and Brain Sciences Unit Volunteers Panel. Participants were eligible if they (a) were 18 to 65 years old; (b) reported no history of mental health, neurological, or psychiatric illness; and (c) had not taken part in similar studies. Before the experiments, participants were informed of the nature of the film content and provided their written and informed consent. The study was approved by the University of Cambridge Psychology Research Ethics Committee (2014/3214). Given an effect size of *d* = 1.05 from Lau-Zhu et al. (2019, Experiment 3), 18 participants per interference group were required for 80% power to attempt to replicate the interference effect on same-day intrusions at a 5% significance level (two-tailed).

### Materials and measures

#### Trauma film

Eleven distinct films with traumatic content were obtained from the public domain. Examples included a girl being injured after a car accident and documentary footage of a famine in Rwanda. Such films have been used extensively in previous studies in this line of work (Holmes et al., 2009; James et al., 2015; Lau-Zhu et al., 2019). The films were presented immediately one after the other, using E-Prime (Version 2.0; Schneider et al., 2002) on a desktop screen (size: 32 × 40 cm; resolution: 1,280 × 1,024 pixels; distance: ≈100 cm from the screen). The soundtrack was played via headphones.

#### Cognitive-interference procedure

A film-reminder task was followed immediately by Tetris game play.

##### Film-reminder task

Eleven replica full-screen stills (one per film clip) were presented for 3 s each against a black background in the same fixed order as in the film. Each still typically depicted the instance before the “worst” moment in the film (Ehlers et al., 2004), such as the face of an injured girl in the car crash (before she was dragged on the road) or a picture of a village in Rwanda (before details of famine were presented). Participants were asked to “sit still and pay close attention to the pictures” with the intention that these memories would be brought to mind. Stills were presented via E-Prime software (Version 2.0; Schneider et al., 2002).

##### Tetris

Tetris is a desktop-based game that consists of different colored and shaped 2-D geometric figures (Blue Planet Software, 2007). Each figure falls from the top of the game screen and can be rotated 90° at a time. Participants had to form full horizontal lines without gaps using these figures. They were trained in and encouraged to use mental rotation to play the game by focusing not just on the figure that is currently falling but also by working out how best to use the three figures shown in a preview, which were due to fall, using mental rotation in their mind’s eye to simulate the fall and best placement (Lau-Zhu et al., 2017, 2019; Rackham & Lau-Zhu, 2021). As game-play scores increased, so did the difficulty level. The use of Tetris is based on the hypothesis that it taxes capacity-limited visuospatial working memory resources (Lau-Zhu et al., 2017) that are needed to hold mental images (Baddeley & Andrade, 2000; Rackham & Lau-Zhu, 2021). Game was played in “marathon” mode (with 15 levels) and sound off.

#### Filler tasks

A 30-min structured break consisted of (a) a knowledge-search task (answering a list of questions using an encyclopedia), (b) a music-rating task, and (c) a second round of the knowledge-search task. Details of these tasks can be found elsewhere (Holmes et al., 2009; Lau-Zhu et al., 2019).^1^

#### Self-report measures

Assessments were conducted for baseline depressive symptoms (Beck Depression Inventory–II; Beck et al., 1996), trait anxiety (State Trait Anxiety Inventory–Trait; Spielberger et al., 1983), trauma history (Traumatic Experience Questionnaire; Foa et al., 1999), and general mental imagery use (Spontaneous Use of Imagery Scale; Nelis et al., 2014). Visual analogue scales (sad, depressed, and hopeless) were used to assess mood change from before to after film viewing, and 11-point Likert scales were used to assess attention paid to the film, personal relevance of the film, diary compliance, and demand ratings (Lau-Zhu et al., 2019).

### Measures of memory of the trauma film

Except for the intrusion diary, tasks were presented using MATLAB (Version 7.8; The MathWorks Inc., Nantucket, MA) and Psychophysics Toolbox (Version 3.0; Brainard, 1997).

#### Vigilance-intrusion tasks

**Fig. 2.**
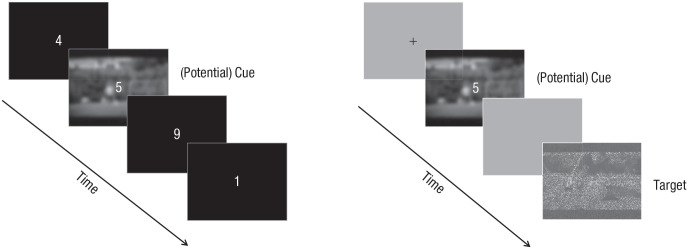
Schematic for the vigilance-intrusion tasks (intrusive memory) and the modified recognition task (voluntary memory). Example trials of the vigilance-intrusion tasks are shown on the left. Participants performed a simple go/no-go task (respond only when the digit “3” appears). A background picture was presented every three trials, mostly depicting landscapes. Additional picture cues were presented infrequently, which were either stills from the trauma films or foil (unseen) films. For the foil-cue version, participants also undertook the task in a different experimental room. An example trial of the modified recognition task is shown on the right. Each trial began with a fixation cross, followed by picture cue (trauma-film cue or foil cue). Then a blank screen appeared, followed by the recognition target. Potential cues were the same in all tasks. In the example, the cue still depicts the moment before the accident when the boy is standing in a football court; the target still depicts the moment when the father cries while seeing the dead body of his son killed by a car (both cue and target belong to the same film sequence). Stills are for illustration and not up to scale; stills in the experiment were in color.

The basic task structure (Fig. 2) was based on previous adaptation (Lau-Zhu et al., 2019, Experiments 2 and 3) of a go/no-go paradigm (Murphy et al., 2013). On each of the 270 trials, a digit (1–9) was presented centrally against a black background for 250 ms, followed by just the background for 1,500 ms. Participants were asked to press “go” only when they saw the digit “3” (11% probability). Background indoor/outdoor scenes (altered with Gaussian Blur 2.0 and not related to the trauma film) were presented every three trials (behind the digit), intended to simulate everyday cues of involuntary memories, which are typically unattended (Berntsen, 2009). Scenes and digits were presented in a fixed randomized order. Viewing distance was 60 cm approximately.

Participants were asked to press an “intrusion” key using the keyboard whenever they experienced an intrusive memory of the film. The number of trauma-film intrusions in this task has shown good test–retest reliability across Day 1 and Day 8 and can predict subsequent diary intrusions (Lau-Zhu et al., 2019).

There were two versions/conditions for this task, each with 11 additional background scenes (also blurred and presented twice each in a fixed randomized order). For the trauma-film-cue condition, scene stills (i.e., film cues) were drawn from the trauma film. These stills, similar to those in the film-reminder task, were also of nonhotspot moments but unlike those in the film-reminder task, depicted different neutral content. Furthermore, the task was delivered in the same room as where the entire experiment took place. In contrast, for the foil-cue condition, additional scenes (i.e., foil cues) were drawn from unseen films that were never shown in the experiment. These unseen films were similar in content and themes to the trauma film and were also drawn from similar sources in the public domain. The foil-cue condition was also completed in a different experimental room to where trauma film was viewed—a similar procedure has been carried out in other memory studies because the experimental context could in itself provide additional cues to promote retrieval (e.g., Kroes et al., 2013). Each condition took approximately 9 min.

#### Modified recognition task

The basic task structure (Fig. 2) was based on an attention-to-memory paradigm in which attentional cues were manipulated before recognition targets (Ciaramelli et al., 2010), building on our previous visual-recognition memory task (Lau-Zhu et al., 2019, Experiments 1 and 2). Our current task involved two sets of stimuli (for cue scenes and target scenes). The cue set had 22 stills (the same 11 cue stills from each of the two vigilance-intrusion conditions). The target set had 236 stills. Half of the target set was taken from the trauma film (i.e., film targets) and half from footage from unseen films (i.e., foil targets). The unseen films were the same ones as in the foil-cue condition of the vigilance-intrusion task. All target stills depicted different moments to those in the cue stills (thus also different from the stills used in the vigilance-intrusion tasks or in the film-reminder task). Target stills were blurred with salt-and-pepper noise (black and white pixels superimposed on the still; Lau-Zhu et al., 2019, Experiment 1) to increase task difficulty and avoid potential ceiling effects.

Each trial began with a blank screen (dark gray background) for 1,500 ms. A cue still was then presented for 1,500 ms. Simultaneously, a digit (1–9) was presented and superimposed in the center of the cue still for 750 ms. Participants were asked to press the “go” key as soon as the digit “3” appeared (this go/no-go component matched the vigilance-intrusion task and required temporal attention during the cue presentation, which could have been ignored otherwise). Afterward, the cue still disappeared, and another blank screen remained for 1,500 ms. Finally, a target still was presented and remained on screen up to 5 s until a response was made. Participants were instructed to decide as quickly and as accurately as possible whether this target still was taken from the trauma film or not (YES/NO) using the keyboard.

Film targets and foil targets were equally paired with film and foil cues. When there was a source match between cue and target (i.e., film cue with film target or foil cue with foil target), both were drawn from the same footage, thus any performance advantage conferred by film cues as opposed to foil cues can be attributed to the mnemonic relationship between cues and targets within the same footage rather than purely a perceptual match (e.g., same color palette). There were therefore four randomly intermixed trial types, obtained by independently crossing trauma-film cues versus foil cues with trauma-film targets versus foil targets. Assignment of target stills to cue type (matching vs. nonmatching cue from the same film/foil source) was counterbalanced across participants. There were 270 trials in total (34 were filler trials using other indoor/outdoor scenes to match the trial number of the vigilance-intrusion tasks and were excluded in the analyses). Trials were presented in a fixed random order. The task took 40 min approximately.^2^

#### Intrusion diary

Participants were instructed to note down any intrusive memory of the film (checking a tick box and writing a brief description) in a daily pen-and-paper tabular diary in each of three time bins per day (Lau-Zhu et al., 2019). Intrusive memories were defined as “visual images, sounds and bodily sensations related to the film,” which could be from “fuzzy and fragmented” to “vivid and as clear as normal vision” and could “pop into mind without one expecting it.” Intrusive memories could include verbal thoughts but only if they had an imagery component as well. Participants were told to exclude deliberate retrieval and to set aside a regular time to complete it. Participants were encouraged to write down each intrusion as soon as it occurred and if this was not possible, to record their intrusions retrospectively once at the end of each time bin (morning, afternoon, and evening). Even if they had zero intrusions, they were instructed to note down “0” and cross out that particular time bin without intrusions. Participants were also asked to write a brief description of the film overleaf so that the content of each intrusion could later be matched back, or not, to a given scene in the film. Only intrusions of the film were not counted. Instructions on how to keep the diary were given orally and also supplemented with a checklist to confirm the participant’s comprehension. Bespoke discussion was given around how to keep and use the diary (e.g., in their bag to ensure they completed it). Participants were given a prepaid envelope to post back the diary upon completion. One week after the single-session experiment, participants were sent a reminder e-mail to return the diary.

### Procedure

The experiment consisted of a single 3-h session. After informed consent, participants completed baseline measures and practiced playing Tetris using mental rotation for 1 min. They then completed mood ratings and viewed the trauma films alone in a darkened room. They were clearly instructed to imagine themselves witnessing the events in the films as if they were actually happening in front of their eyes (to encourage immersion in film viewing). After the films, participants completed mood ratings again and additional self-report measures of attention and personal relevance and completed a 30-min structured break (with filler tasks). Following the break, participants were randomly assigned to one of two interference groups. In the reminder-plus-Tetris group, participants underwent the film-reminder task and then played Tetris uninterrupted for 10 min (with instructions to use mental rotation). In the reminder-only group, participants underwent the film-reminder task and sat quietly for 10 min, doing nothing else.

Then all participants completed the memory measures in a fixed-order design. They were first given instructions to complete the vigilance-intrusion tasks, followed by a short practice. They completed the foil-cue condition first (went into a different room) followed by the trauma-film-cue condition (returned to the original room) to prevent potential carryover effects of the trauma-film cues. Afterward, they completed the modified recognition task following a short practice.

Finally, participants were given instructions to complete the diary and return these by free post upon completion. To enhance the likelihood of diaries being returned, our diary protocol included (a) emphasis on the importance of returning the diary for the study results, communicated verbally and later in writing in a reminder e-mail; (b) explanation that they would be compensated only after diaries were completed and returned; and (c) a reminder e-mail to return the diary sent a week after the experimental session. Upon return of the diary, participants were compensated and then debriefed by e-mail (with the option of a phone call if they wished, although this option was not taken up).

### Statistical analyses

No univariate outliers (> 3 *SD* from the mean; Tabachnick & Fidell, 1996) were identified within any experimental condition. Performance above chance was assessed with one-sample *t* tests. Interference groups were compared with independent sample *t* tests, and Levene’s statistic was used to test homogeneity of variance. Analyses of variance (ANOVAs) with repeated measures were used when both within-groups and between-groups variables were included. Unless otherwise stated, we used a two-tailed α level of .05. Nonparametric tests were used when assumptions of parametric tests were violated; results converged with those of the parametric tests, so we report results from parametric tests below. Analyses were performed using IBM SPSS (Version 25.0) and MATLAB 2016 (The MathWorks Inc., Nantucket, MA).

## Results

### Randomization, manipulation check for film viewing and diary, and demand ratings

Descriptive statistics are presented in Table 1. The sample’s ethnic makeup, as described by participants, was 74% White, 20% Chinese, and 6% Asian (Indian, Pakistani). Employment statuses were described as 75% students, 22.2% in full-time employment, and 2.78% unemployed. Interference groups did not differ at baseline in terms of gender, χ^2^ = 1.33, *df* = 1, *p* = .248, age, depressive symptoms, trait anxiety, number of previous traumatic events, and general use of imagery, *t*(34)s < 1.39, *p*s > .175. There was a main effect of time on mood ratings, consistent with an increase in negative mood from before to after film viewing, *F*(1, 34) = 54.95, *p* < .001, η_*p*_² = .618, and as expected, neither the main effect of interference group, *F*(1, 34) < 1, nor the interaction of group and time, *F*(1, 34) = 1.38, *p* = .249, was significant. Ratings for attention paid to the film, personal relevance of the film, and diary accuracy and demand ratings were also not significantly different between interference groups, *t*(34)s < 1.50, *p*s > .144.

**Table 1.** Baseline, Manipulation Check for Film Viewing and Diary, and Demand Ratings by Interference Group

Characteristic	Reminder plus Tetris (*n* = 18)	Reminder only (*n* = 18)
Gender		
Female (*n*)	12	15
Male (*n*)	6	3
Age (years)	25.11 (2.40)	23.56 (4.12)
Measures		
BDI-II	5.00 (6.16)	4.39 (3.26)
STAI-T	35.89 (11.32)	35.44 (8.31)
TEQ	0.72 (0.96)	0.39 (0.70)
SUIS	39.67 (7.51)	39.44 (9.35)
Prefilm negative mood	2.17 (3.56)	3.30 (4.10)
Postfilm negative mood	9.54 (7.28)	8.66 (5.54)
Attention paid to the film	9.39 (0.70)	9.44 (0.62)
Personal relevance of film	3.89 (2.30)	4.78 (2.49)
Diary accuracy	9.44 (0.98)	8.89 (1.23)
Demand ratings	−2.89 (2.70)	−1.22 (4.48)

Note: Values are means with standard deviations in parentheses unless otherwise noted. Each negative mood is a composite score summing three visual analogue scales on sad, depressed, and hopeless moods. BDI-II = Beck Depression Inventory–II (Beck et al., 1996); STAI-T = State Trait Anxiety Inventory–Trait (Spielberger et al., 1983); TEQ = Traumatic Experience Questionnaire (Foa et al., 1999); SUIS = Spontaneous Use of Imagery Scale (Nelis et al., 2014).

### Aim 1: replication of previous findings on intrusive-memory reduction

#### Early/laboratory intrusions

The mean number of “early” intrusions in the vigilance-intrusion task with trauma-film cues (Table 2) was similar to previous versions used in Lau-Zhu et al. (2019, Experiments 2 and 3). Note that our first prediction was confirmed in that we replicated prior findings of fewer intrusions in the reminder-plus-Tetris group than in the reminder-only group, *t*(34) = 2.88, *p* = .007, *d* = 0.96 (Table 2). That is, an interference effect was established.

**Table 2.** Key Outcomes on Measures of Intrusive Memories and of Voluntary Memory by Cue Type and Interference Group

Measure	Reminder plus Tetris (*n* = 18)		Reminder only (*n* = 18)	
	*M*	*SD*	*M*	*SD*
Vigilance-intrusion tasks				
Trauma-film cues	7.56	6.87	17.94	13.69
Foil cues	4.39	3.63	11.39	14.33
Intrusion diary				
1-week period	2.56	3.75	5.83	5.08
Modified recognition task				
Trials with trauma-film cues				
Hits	41.22	4.61	45.11	5.78
FA	17.44	9.39	15.11	7.45
Accuracy	0.41	0.17	0.51	0.17
Trials with foil cues				
Hits	41.44	7.52	42.28	6.12
FA	19.11	9.92	16.00	8.33
Accuracy	0.38	0.21	0.46	0.17

Note: Outcome for intrusive memories is the number of occurrences. Recognition accuracy was calculated by subtracting the FA rate (FA / [FA + CR]) from the hit rate (Hit / [Hit + Miss]). FA = false alarms; CR = correct rejection.

#### Intrusion diary

All diaries (100%) were completed and returned by post. Two researchers scored all diaries independently. Interclass correlation was 1.00, suggesting perfect agreement (using two-way mixed-effects model, consistency, single measure; McGraw & Wong, 1996). Virtually all intrusive images were matched to the trauma film (98%), such as “image of a razor cutting across shaving neck” and “imagined incision of bone.” The mean (Table 2) and range for intrusion frequency in the reminder-only control group (1–16) are consistent with previous studies (Holmes et al., 2009; Lau-Zhu et al., 2019, Experiments 1 and 2). Again replicating previous studies (Holmes et al., 2009; Holmes, James, et al., 2010; Lau-Zhu et al., 2019), we found that the reminder-plus-Tetris group had fewer diary intrusions than the reminder-only group, *t*(34) = 2.20, *p* = .034, *d* = 0.73 (Table 2).

### Aim 2: testing competing accounts for the intrusive or voluntary memory dissociation

#### Vigilance-intrusion tasks

The 2 (group: reminder plus Tetris vs. reminder only; between groups) × 2 (cue type: trauma-film cues vs. foil cues; within groups) mixed-model ANOVA on intrusions revealed a significant main effect of cue type, *F*(1, 34) = 19.61, *p* < .001, η_*p*_² = .366 (90% confidence interval CI = [0.15, 0.52]); there were more intrusions overall after trauma-film cues (*M* = 12.75, *SD* = 11.90) than after foil cues (*M* = 7.89, *SD* = 10.90), demonstrating that our manipulation worked as intended.

The main effect of interference group was also significant, *F*(1, 34) = 6.64, *p* = .014, η_*p*_² = .163 (90% CI = [0.02, 0.34]), consistent with the results for the trauma-film-cue condition alone reported in Aim 1 above and confirming that the reminder-plus-Tetris group (*M* = 5.92, *SE* = 2.39) reported fewer intrusions overall than the reminder-only group (*M* = 14.67, *SE* = 2.39).

However, the Group × Cue Type interaction was not significant, *F*(1, 34) = 2.38, *p* = .132, η_*p*_² = .065 (90% CI = [0, 0.22]). Thus, there was no evidence that the effect of reminder-plus-Tetris in reducing intrusion frequency depended on whether cues from the same film were present to trigger associated memories.

#### Modified recognition task

We calculated recognition accuracy as in Lau-Zhu et al. (2019, Experiments 1 and 2) by subtracting the false alarm (FA) rate (calculated as FA / [FA + CR], where CR = correct rejections) from the hit rate (Hit / [Hit + Miss]; see Table 2). Recognition accuracy was above chance for targets that followed trauma-film cues and also for those that followed foil cues, *t*(17)s > 7.59, *p*s < .001, *d*s > 1.95.

The 2 (group: reminder plus Tetris vs. reminder only; between groups) × 2 (cue type: trauma-film cues vs. foil cues; within groups) mixed ANOVA on recognition accuracy revealed a main effect of cue type that approached significance, *F*(1, 34) = 3.25, *p* = .080, η_*p*_² = .087 (90% CI = [0, 0.25]). This was in the predicted direction: There was higher recognition accuracy following trauma-film cues than following foil cues, suggesting that our manipulation again worked as intended.

The main effect of interference group, however, was not significant, *F*(1, 34) = 2.40, *p* = .131, η_*p*_² = .066, 90% CI = [0, 0.22] (reminder plus Tetris: *M* = 0.40, *SE* = 0.04; reminder only: *M* = 0.48, *SE* = 0.04), nor was the Group × Cue Type interaction, *F*(1, 34) = 0.59, *p* = .447, η_*p*_² = .017, 90% CI = [0, 0.14]. This replicates prior findings (Lau-Zhu et al., 2019) that reminder plus Tetris does not affect measures of voluntary memory (the selective-interference effect) and goes further to suggest that this lack of an effect applies even when the recognition memory test is modified to include an associative component.

## Discussion

We set out to test whether intrusive memories can be reduced (by a subsequent cognitive interference procedure) while sparing voluntary memory—a selective-interference effect (Lau-Zhu et al., 2019)—or whether the apparent dissociation simply reflects differences in how the memories are measured. More specifically, we hypothesized that there was a difference in the associative component of the measures in that measures of intrusive memories more readily probe associations between a trigger cue (e.g., “a picture of a boy playing football”) and a target hotspot memory (e.g., imagery of “the same boy being killed by a car”) than do typical measures of voluntary memory, which have tended to rely on recognition memory for individual (nonassociative) elements of an event. Therefore, if an interference task were to disrupt associations between elements, then this disruption would be more apparent in measures of intrusive memories than in measures of voluntary recognition memory.

To test this possibility, we administered an interference procedure (reminder plus Tetris) shortly after asking participants to watch a trauma film and examined its effects on subsequent memory of that film, as assessed by memory measures that differed in intentionality (i.e., involuntary vs. voluntary) and cue type (with vs. without trauma-film cues). Replicating previous studies (Badawi et al., 2020; Holmes et al., 2009; Holmes, James, et al., 2010; Lau-Zhu et al., 2019), we found that reminder plus Tetris reduced memory on all measures of involuntary memory (i.e., intrusion rates, including on an additional diary measure) but not on any voluntary recognition accuracy. More importantly, this pattern of results occurred regardless of whether recognition trials (or intrusion condition) were accompanied by associative cues from the same trauma film (vs. from unseen films).

We found the associative memory did not appear to contribute to the dissociation between intrusive and voluntary memory. To test the potential contributions of associative memory, we compared conditions with trauma-film cues and without trauma-film cues in both a vigilance-intrusion task and a modified recognition task. As expected, conditions with trauma-film cues (e.g., blurred picture of “a boy in a football pitch”) tended to enhance retrieval across both memory types: higher intrusion rates in the vigilance-intrusion task (e.g., more intrusive mental images of “the same boy being killed by a car”) and a tendency to improve accuracy in the modified recognition task (e.g., better recognition of a still depicting a scene in which “the dad is crying over the boy’s dead body”). These results confirmed that our manipulation of associative cues sufficiently increased accessibility of involuntary/intrusive memories (Berntsen, 2009; Ehlers & Clark, 2000; Schlagman & Kvavilashvili, 2008) and recognition memory (Ciaramelli et al., 2010). Nevertheless, and critically, associative-memory cues did not moderate the selective-interference effect and thus were unlikely to account for it.

To our knowledge, our design provided the closest head-to-head comparison to date between intrusive memory and voluntary memory in the trauma-film literature (Brewin, 2014; James et al., 2016; Lau-Zhu et al., 2018). In addition to matching in terms of associative-memory cues (i.e., presence and absence of them), intrusion and recognition tasks were also closely matched in terms of context (both in the laboratory), time of testing (Day 1), as well as attentional demands (both had a go/no-go element during cue exposure and initially unattended triggering cues), facilitated by the use of a laboratory analogue of the diary employed here—the vigilance-intrusion task. This closed matching extends our recent work ruling out other retrieval factors, such as cue overlap (Lau-Zhu et al., 2019, Experiment 1), attentional capture (Lau-Zhu et al., 2019, Experiment 2), retrieval load (Lau-Zhu et al., 2019, Experiments 3), and retention interval (James et al., 2015, Experiments 1 and 2; Lau-Zhu et al., 2019, Experiment 2). The most parsimonious explanation for our selective interference remains that involuntary/intrusive memories and voluntary memories, at least in the context of trauma, are supported by different memory systems. This separation would allow memory traces in one system to be disrupted by an interfering task without affecting traces in the other. This rationale is consistent with separate-trace memory accounts, the most prominent one being the dual-representation theory of PTSD (Bisby & Burgess, 2017; Brewin, 2014; Brewin et al., 1996).

Why can (intrusive) memories for trauma materials behave differently from memories for nontraumatic, ordinary events? The differential predictions tested here—between single-trace and separate-trace accounts—have been part of a long-standing debate in the PTSD literature, which has remained unresolved for more than two decades, at least within research using the trauma-film paradigm (James et al., 2016; Lau-Zhu et al., 2019). The difficulty in resolving this debate has been exacerbated by the methodological challenges involved in directly pitting these accounts against each other. Many separate-trace conceptualizations are anchored in clinical psychology in which understanding the clinical phenomena is central. Intrusive images are key to the patient experience of traumatic events yet notoriously difficult to “pin down” given their probabilistic occurrence. Clinical researchers thus have relied on more naturalistic techniques (e.g., diaries) to understand these memories. In contrast, many single-trace conceptualizations are grounded in cognitive psychology, in which a greater emphasis is placed on experimental control. It is not surprising, then, that voluntary—rather than involuntary—forms of memory have been the main foci for basic researchers.

To resolve the theoretical debate, discrepancies in the associated methods first need to be addressed. This is the challenge we took in our current study, during which intrusions were brought “into the lab” to allow greater experimental control and the recognition test was modified to incorporate a potentially key feature of intrusive memory recall in everyday context (involving associate memory). The current findings of selective interference, despite our methodological innovation, provide greater confidence that separate-trace accounts better explain why (intrusive) trauma memories can behave differently to nontraumatic, ordinary memory, that is, the existence of separate memory traces for traumatic or highly aversive materials. It is critical to understand when involuntary memory and voluntary memory for the same materials can be dissociated and when and how they may influence each other (e.g., Herz et al., 2020). However, this will no doubt remain a controversial account, not least because of being less parsimonious. We need continuing refinement of memory measures for further rigorous demonstrations of the intrusive/involuntary and voluntary dissociation and application of these measures to memoranda other than trauma films, including to memory paradigms that traditionally do not include a measure of involuntary recall.

What are the practical implications of our findings, such as for clinical and forensic psychologists? Knowing that a modification in voluntary memory is neither equivalent nor necessary for changes in intrusive memories of traumatic events opens up avenues for improved interventions. First, clinicians would benefit from knowing the nuanced profile of how an intervention, whether psychological or pharmacological, affects both intrusive and voluntary aspects of trauma memory. If there is a dissociation between involuntary memory and voluntary memory, then one cannot always extrapolate intervention effects on nonintrusive/voluntary memory to changes in intrusive imagery of the same traumatic events. Therefore, clinical developments in this area ought to always include measures of intrusive memories in their key outcomes to facilitate translational research and to maximize their impact on the sort of memory that is central to the patient’s distress (Iyadurai et al., 2019). In addition, one cannot assume that after an intervention, voluntary memory always remains intact either, which is important to consider to assess the relative costs and benefits of the intervention (Holmes, Sandberg, & Iyadurai, 2010).

Second, the cognitive procedure examined in this line of work (involving reminder procedures and cognitive tasks) represents a promising means to exert selective interference in the wake of trauma (Singh et al., 2020). One advantage is that this simple cognitive-task approach may be developed in a manner to maximize safety and minimize distress while doing the intervention, which is important given that particular clinical approaches have been shown to worsen trauma symptoms (Rose et al., 2002) and that approaches that require talking about the trauma in detail are not tolerated by all patients (Hoge & Chard, 2018; National Institute for Health and Clinical Excellence, 2018). Initial proof-of-concept studies using the reminder-plus-Tetris procedure are promising in preventing intrusive symptoms within the first week after trauma (Horsch et al., 2017; Iyadurai et al., 2018; Kanstrup, Singh, et al., 2021) and in reducing more persistent intrusive symptoms (Iyadurai et al., 2020; Kanstrup, Knotio, et al., 2021), including in established PTSD (Kessler et al., 2018). In these translation studies, the necessary reminder cue (James et al., 2015, Experiment 2; Lau-Zhu et al., 2019, Experiment 3) was adapted to each clinical context, with variations such as a brief hotspot verbal reminder (Iyadurai et al., 2018; Kanstrup, Kontio, et al., 2021; Kanstrup, Sing, et al., 2021), a context reminder (Horsch et al., 2017), or a written reminder (Kessler et al., 2018). A related procedure using Tetris is also being explored to modulate distressing imagery that result from indirect exposure to collective trauma via the media in a real-world context (Rackham & Lau-Zhu, 2021). We note that there are likely to be many routes by which intrusive trauma memory may be modulated, including a cognitive-task procedure (Singh et al., 2020), traditional psychological therapies, and pharmacological and neuromodulation, all of which may further evolve.

Third, our data have relevance for the legal context, too. Trauma survivors may want to remain able to provide accurate reports of traumatic incidents at will even if their intrusions are eliminated. Moreover, as often arises, for example, in asylum and refugee cases, decision makers typically assume that trauma should be always well remembered (Herlihy et al., 2010), whereas our data suggest that one cannot judge the level of recall of involuntary memory on the basis of the level of recall in voluntary memory, given its possible fractionation.

There are some caveats with our experimental design. There may be effects of test order because we tested intrusions before recognition. This decision was informed by evidence indicating that measures of intrusions can be influenced by preceding tasks probing for voluntary memory (Krans et al., 2009). Although the reverse is also possible, that assessing intrusions effects may have somehow “masked” effects of interference on subsequent recognition effects, we do not think this is likely. For the vigilance-intrusion task, the foil-cue condition always preceded the trauma-film-cue condition. This was again intended to avoid carryover effects from associative memories triggered by trauma-film cues into the foil-cue condition. It is possible that intrusion frequency in this type of task changes with the passage of time and that this caused the main effect of cue type. This again seems unlikely, however, because one would expect a decline in intrusion frequency with time, yet more intrusions occurred in the condition with trauma-film cues, which were administered later in time. Results in the intrusion tasks seem unlikely to be due to the lack of counterbalancing, but between-groups designs might be considered in future studies to address this issue. It is also possible that our vigilance-intrusion task does not fully capture the situations under which intrusions occur in real life. Although our go/no-go task is designed to simulate attentional demands of everyday life (e.g., shopping or reading) during which intrusions spontaneously occur, it may not match other cognitive conditions that are conducive to intrusions. Furthermore, the cues we used (blurred stills) may not correspond to the cues that trigger intrusions in real life; clinical accounts (Ehlers & Clark, 2000) suggest that potent cues can be highly idiosyncratic (e.g., a simple patch of red color triggering an intrusive memory of blood).

Future studies seeking to test replication may wish to increase sample size. The main effect of cue type within the modified recognition memory task, although in the predicted direction and meeting one-tailed threshold, was not significant at a two-tailed threshold and thus may have lacked power. For the interactions between interference group and cue type (within the vigilance-intrusion data and the recognition data), we had sufficient power to detect standardized medium to large effects (Cohen’s *f* of .25 or .40; Cohen, 1988) but not to detect potential small interaction effects (Cohen’s *f* of .10; Cohen, 1988). See also the Supplemental Material available online. It is difficult to ascertain what effect size one would expect for such interactions given the novelty of our work (simultaneously looking at interference, cue type, and memory type), but we hope that the effect sizes reported here will be helpful for future studies.

Interventions after traumatic events are sometimes reported in the media as if they provide full deletion of trauma memories (e.g., as depicted in the movie *Eternal Sunshine of the Spotless Mind*; Gondry, 2004). A more likely and desirable approach is to selectively modify one clinically distressing aspect of trauma memory (Elsey & Kindt, 2016; Visser et al., 2018)—such as its intrusiveness—without causing complete forgetting of the trauma (Holmes, Sandberg, & Iyadurai, 2010; Lau-Zhu et al., 2019). One way in which involuntary aspects of memory are amenable to selective modification—independently of voluntary aspects—is through a simple-to-deliver procedure involving a memory reminder cue and a cognitive task (e.g., mental rotation plus Tetris game play). This approach holds potential for a new generation of effective, safe, and mechanistically informed memory therapeutics that are less aversive for patients than current trauma-focused psychological interventions and are also more readily disseminated. The theoretical possibility of a separate, declarative memory system for traumatic events—as raised by our data—illustrates the need for conventional memory accounts to better accommodate intrusive phenomena and, crucially, underscores how fundamental research also stands to gain from insights in clinical psychopathology.

## Supplemental Material

sj-pdf-1-cpx-10.1177_2167702621998315 – Supplemental material for Selectively Interfering With Intrusive but Not Voluntary Memories of a Trauma Film: Accounting for the Role of Associative Memory Click here for additional data file. Supplemental material, sj-pdf-1-cpx-10.1177_2167702621998315 for Selectively Interfering With Intrusive but Not Voluntary Memories of a Trauma Film: Accounting for the Role of Associative Memory by Alex Lau-Zhu, Richard N. Henson and Emily A. Holmes in Clinical Psychological Science

## Transparency

*Action Editor:* Colin MacLeod

*Editor:* Kenneth J. Sher

*Author Contributions*

A. Lau-Zhu developed the study concept. All authors contributed to the study design. A. Lau-Zhu programmed the experimental tasks and performed data collection, data analyses, and interpretation under the supervision of R. N. Henson and E. A. Holmes. A. Lau-Zhu drafted the manuscript, and E. A. Holmes and R. N. Henson provided critical revisions. All of the authors approved the final manuscript for submission.

**Open Practices:** All data and materials have been made publicly available via OSF and can be accessed at https://osf.io/uymdw. This article has received badges for Open Data and Open Materials. More information about the Open Practices badges can be found at https://www.psychologicalscience.org/publications/badges.

## Notes

1. This methodology was selected for consistency with prior studies using the same 30-min postfilm delay to ensure equivalent activities across participants, and tasks were selected on the basis of the assumption that they were low in visuospatial demands, so we can be more confident that any interference effect attributable to a “visuospatial” task would be specific to Tetris game play.

2. There was an additional set of filler trials (created using two cue stills and 34 target stills, all depicting various indoor/outdoor scenes) that were excluded from all analyses.
