# Supplementary material for: Selectively Interfering With Intrusive but Not Voluntary Memories of a Trauma Film: Accounting for the Role of Associative Memory
Source: Clin Psychol Sci. 2021 May 6;9(6):1128–43. doi: 10.1177/2167702621998315 (PMC8579330; doi:10.1177/2167702621998315)
Supplement: sj-pdf-1-cpx-10.1177_2167702621998315 – Supplemental material for Selectively Interfering With Intrusive but Not Voluntary Memories of a Trauma Film: Accounting for the Role of Associative Memory [file sj-pdf-1-cpx-10.1177_2167702621998315.pdf]

Supplemental Material

Lau-Zhu, A., Henson, R.N., & Holmes, E.A. Selectively Interfering with Intrusive but not Voluntary Memories of a Trauma Film: Accounting for the Role of Associative Memory

Bayesian Analyses

As exploratory analyses, we calculated a Bayes Factor to check whether there was sufficient evidence to support the null of no interference on voluntary memory overall. Using Gönen's method (Gönen et al., 2005) where the alternative hypothesis was based on the size of interference on total laboratory intrusions collapsed across both task conditions (d = .86), the associated Bayes Factor in favour of the null on overall recognition, BF01, was 3.69, which is considered substantial (Dienes, 2011; Jeffreys, 1998), meaning the data favoured the null over three times more than the alternative.

We also used a Bayesian approach (Dienes, 2014; Greve et al., 2014) to explore the null effects on the key interactions between interference group and cue type, within both the vigilance-intrusion data and the recognition data. We compared the null with the alternative that the size of interference (i.e., group difference in memory) observed in the trauma-film cue condition would be completely absent in the foil cue condition. For the vigilance-intrusion data, this means an interaction value of 3.38 with SE of 2.19, and a rectangular prior with 0 as the minimum and 10.38 as the maximum. For the recognition data, this means an interaction value of 3.05 with SE of 3.96, and a rectangular prior with 0 as the minimum and 3.89 as the maximum. This approach yielded BF01 = 1.63 for the vigilance-intrusion data,

and 1.25 for the recognition data, indicating that the both group of data were insensitive from a Bayesian perspective (Dienes, 2011; Jeffreys, 1998).

References for the Supplemental Material

Dienes, Z. (2011). Bayesian versus orthodox statistics: Which side are you on? *Perspectives on Psychological Science*, *6*(3), 274–290. https://doi.org/10.1177/1745691611406920

Dienes, Z. (2014). Using Bayes to get the most out of non-significant results. *Frontiers in Psychology*, *5*, 781. https://doi.org/10.3389/fpsyg.2014.00781

Gönen, M., Johnson, W. O., Lu, Y., & Westfall, P. H. (2005). The bayesian two-sample t-test. *The American Statistician*, *59*(3), 252–257. https://doi.org/10.1198/000313005X55233

Greve, A., Cooper, E., & Henson, R. N. (2014). No evidence that “fast-mapping” benefits novel learning in healthy older adults. *Neuropsychologia*, *60*(1), 52–59. https://doi.org/10.1016/j.neuropsychologia.2014.05.011

Jeffreys, H. (1998). *Theory of probability* (3rd ed.). Clarendon Press.
